# Halophytes Differ in Their Adaptation to Soil Environment in the Yellow River Delta: Effects of Water Source, Soil Depth, and Nutrient Stoichiometry

**DOI:** 10.3389/fpls.2021.675921

**Published:** 2021-06-01

**Authors:** Tian Li, Jingkuan Sun, Zhanyong Fu

**Affiliations:** ^1^Shandong Key Laboratory of Eco-Environmental Science for Yellow River Delta, Binzhou University, Binzhou, China; ^2^School of Chemical and Environmental Engineering, China University of Mining and Technology, Beijing, China

**Keywords:** Yellow River Delta, halophyte, stable isotope, water use, stoichiometry characteristics

## Abstract

The Yellow River Delta is water, salt, and nutrient limited and hence the growth of plants depend on the surrounding factors. Understanding the water, salt, and stoichiometry of plants and soil systems from the perspective of different halophytes is useful for exploring their survival strategies. Thus, a comprehensive investigation of water, salt, and stoichiometry characteristics in different halophytes and soil systems was carried out in this area. Results showed that the oxygen isotopes (δ^18^O) of three halophytes were significantly different (*P* < 0.05). *Phragmites communis* primarily used rainwater and soil water, while *Suaeda salsa* and *Limonium bicolor* mainly used soil water. The contributions of rainwater to three halophytes (*P. communis*, *S. salsa*, and *L. bicolor*) were 50.9, 9.1, and 18.5%, respectively. The carbon isotope (δ^13^C) analysis showed that *P. communis* had the highest water use efficiency, followed by *S. salsa* and *L. bicolor*. Na^+^ content in the aboveground and underground parts of different halophytes was all followed an order of *S. salsa* > *L. bicolor* > *P. communis*. C content and N:P in leaves of *P. communis* and N content of leaves in *L. bicolor* were significantly positively correlated with Na^+^. Redundancy analysis (RDA) between plants and each soil layer showed that there were different correlation patterns in the three halophytes. *P. communis* primarily used rainwater and soil water with low salt content in 60–80 cm, while the significant correlation indexes of C:N:P stoichiometry between plant and soil were mainly in a 20–40 cm soil layer. In *S. salsa*, the soil layer with the highest contribution of soil water and the closest correlation with the C:N:P stoichiometry of leaves were both in 10–20 cm layers, while *L. bicolor* were mainly in 40–80 cm soil layers. So, the sources of soil water and nutrient of *P. communis* were located in different soil layers, while there were spatial consistencies of soils in water and nutrient utilization of *S. salsa* and *L. bicolor*. These results are beneficial to a comprehensive understanding of the adaptability of halophytes in the Yellow River Delta.

## Introduction

Soil salinity is a major abiotic stress affecting plant growth and productivity throughout the world (Akramkhanov et al., 2011). About 1/10 of the total dry land surface on the earth suffers from salinity problems (Pan et al., 2013). Currently, the saline soil resources in China account for 4.88% of the country’s available land area, and the area of salinized land in the Yellow River Delta is as high as 180,000 hm^2^ (Lim et al., 2017; Xia et al., 2019). Halophytes are plants that have the ability to survive in a saline environment, and are a typical form of vegetation in coastal ecosystems (Kumari et al., 2015). Halophytes have formed a series of physiological salt resistance characteristics in the process of adapting to a saline habitat, and have a stronger ability to overcome salt stress and ion toxicity than non-halophytes. Generally, halophytes are classified into three groups according to their main salt resistance mechanisms (Flowers and Colmer, 2008; Zhang and Shi, 2013). Halophytes can increase ground cover to reduce evaporation, conserve water and soil, improve saline-alkali land, and protect beach and embankment. At the same time, a large area of beach formed by halophytes provides a habitat for all kinds of animals, thus improving the ecological environment (Curado et al., 2014). Studies have shown that planting halophytes on salinity soil can improve soil physical and chemical properties, and have great potential in relieving the pressure of cropping systems and restoring degraded lands (Lei et al., 2018). Therefore, it is of great ecological and economic significance to study the adaptability of halophytes. The adaptability of halophytes is reflected not only in their salt tolerance potential, but also in their ability to utilize water and nutrients.

Water is one of the principle factors limiting plant growth, species diversity, and vegetation distribution (Zhu et al., 2016). The stable isotope technique has become one of the most important techniques in the field of water science. This technique not only has good biological safety, but also overcomes the time and space constraints of traditional research methods (Arslan et al., 1999; Kübert et al., 2020). Therefore, it greatly promotes the research of water source (Yang et al., 2011; Wu et al., 2014; Tetzlaff et al., 2021), water use efficiency (Gouveia et al., 2019; Pan et al., 2020), plant water stress (Zhang et al., 2014), and water use strategy (Eggemeyer et al., 2009; Kanduc et al., 2012; Zhai et al., 2016). At present, stable isotope technology has been widely used in the field of ecology, such as desert, forest, and farmland ecosystems (Cao et al., 2002; Wang et al., 2010; Zhu et al., 2020). Studies have shown that the determination and analysis of stable carbon isotope (δ^13^C) among different species of leaves is an extensive and effective technical means in the study of the difference of water use and water stress adaptation of plants in a period of time (Arslan et al., 1999; Pan et al., 2020), and it is more accurate to use stable oxygen isotope (δ^18^O) as the main tracer index in the study of water use sources of halophytes (Ellsworth and Williams, 2007). The water use source, water contribution, and water use efficiency of plants will change due to the influence of the external environment (such as precipitation, drought, salinity, etc.), and these changes in water use characteristics reflect the adaptability of plants (Chen et al., 2017; Pan et al., 2020; Cui et al., 2021). Therefore, using δ^18^O and δ^13^C stable isotope technologies to clarify the above water use characteristics is helpful to understand the water adaptation of plants.

Carbon (C), nitrogen (N), and phosphorus (P) in plants interact with each other, and there are significant correlations between C, N, and P contents in plants (Schindler, 2003). The alteration of plant stoichiometry can reveal the status of nutrient uptake and utilization by different plants and the restrictive relationship among different nutrients. It is an important index to judge whether plants can renew themselves and recycle nutrients (Crous et al., 2019). Paying attention to the changes in stoichiometry of environment and plants can not only determine the nutrient limiting factors of plant growth, but also provide an important supplement for exploring the relationship between ecosystem stoichiometry and plant function or environmental adaptation mechanism (Cao and Chen, 2017). For instance, studies have shown that the N:P and C:P ratios between soil and plant tissues were strongly correlated, and the plant C:N and C:P ratios responded differently at different nutrient conditions (Cao and Chen, 2017; Wang Z. et al., 2018). On the contrary, Wang et al. (2015) found that leaf C:N, C:P, and N:P were hardly affected by soil features, and there were few correlations with soil nutrition. There are various factors affecting the stoichiometry of plants, such as interactions between leaf traits and soil nutrients, variations in plant-type, and possibly species-level interactions, etc (Cao and Chen, 2017). Thus, a more comprehensive understanding of nutrient utilization would include not only determining the stoichiometry in plants but also those in soil and soil microbial biomass (Li et al., 2012). The carbon: nitrogen: phosphorus (C:N:P) ratio in soil directly reflects soil nutrition and indirectly serves as an indicator of plant nutritional status (Elser et al., 2010). The vegetation in the Shell Dike Island is mainly composed of different halophytes. *Suaeda salsa*, *Limonium bicolor*, and *Phragmites communis* are typical representatives widely distributed in this coastal ecosystem (Tian et al., 2011). Therefore, studying of the stoichiometry of plants and soil, and the correlations between them is helpful to better understand the adaptability of halophytes in the Yellow River Delta.

The Shell Dike Island in the Yellow River Delta is salt-stressed. Fresh water and nutrients are deficient in this area. Halophytes need to change their physiological and biochemical behavior to survive (Drake and Franks, 2003). In recent years, numerous studies have focused on the plant salt tolerance (María et al., 2005; Flowers et al., 2015, 2019), plant water use (Cui et al., 2017; Ferrio et al., 2020; Pan et al., 2020), and plant stoichiometry (Wang et al., 2015; Cao and Chen, 2017; Shang et al., 2018; Crous et al., 2019). However, comprehensive investigation of water, salt, and stoichiometry characteristics in different halophytes and soil systems is scarce. Hence, a study was carried out to investigate the δ^18^O stable isotope, C:N:P stoichiometry, and salt heterogeneity in selected halophyte species (*S. salsa*, *P. communis*, *L. bicolor*) and soil systems in the heterogeneous habitat of Shell Island. The objectives of the present study are to: (1) clarify the characteristics of water use, salinity, and C:N:P stoichiometry of different halophytes, (2) estimate the correlations of water, salt, and stoichiometry in three different halophytes and soil, and thus to better understand the adaptation of halophytes to the coastal environment. The results may enrich the understanding of the “salt-water- stoichiometry” relationship theory of different halophytes and will provide a theoretical basis for coastal vegetation restoration.

## Materials and Methods

### Survey of the Research Area

The study area is located in Wudi County, Shandong Province, the Shell Dike Island (38°18′N, 117°54′E) of the Yellow River Delta. The region belongs to the warm temperate East Asian monsoon continental semi-humid climate zone, with an average annual precipitation of 550 mm. Rainfall is concentrated in June to September (Zhu et al., 2016).

### Plot Setting and Sample Collection

According to the characteristics of soil quality, geomorphology, and land use types in Shell Dike Island of the Yellow River Delta, one sample line was set along the Shell Dike Island. Three points were set on the sample line, the interval between each point was not less than 1 km, three plots of 5 m × 5 m were set in each point, *S. salsa*, *P. Communis*, and *L. bicolor* species and soil samples were collected.

The soil samples were collected in June by using a 4.5 cm diameter special earth drill in five soil depths: 0–10 cm, 10–20 cm, 20–40 cm, 40–60 cm, and 60–80 cm. Three groups of parallel samples were taken. One part of the sample was packed in a self-sealing bag, the other part was immediately packed in a glass bottle with a plug and sealed with parafile. In each sample plot, the mature corked stems of three to four uniform-growing *S. salsa*, *P. Communis*, and *L. bicolor* were collected, respectively. The standard barrel of meteorological station was used to collect rainwater.

The representative homogeneous communities of *S. salsa*, *P. Communis*, and *L. bicolor* were selected. Leaves with sufficient illumination were also selected and then they were mixed into a sample bag with good air permeability. Leaves were dried in 105°C for 20 min, then they were transferred to 70°C for 48 h. The drying material was grinded and selected by 80 mesh sieving.

### Sample Extraction and Determination

All samples were used to extract the water from xylem and soil by vacuum freeze extraction technology (Kanduc et al., 2012). The isotope values of the extracted samples were determined by liquid water isotope analyzer (LWIA, DLT-100, LGR, United States). Hydrogen and oxygen stable isotope ratio: δX(‰) = (*R*_sample_ /R_standard_-1) × 1000, in which R_sample_ is the ratio of heavy to light isotope abundances of elements in samples (H, O)_sample_ and *R*_standard_ is the ratio of stable isotope abundances of international common standards (H, O stable isotopes using v-SMOW) (Einbond et al., 1996). The analytical error for δ^2^H and δ^18^O were ±1.0‰ and ±0.3‰, respectively. The standard sample LGR4E (LGR, United States) was used as the quality control standard to test the stability and accuracy of data analysis. By substituting the average value of the LGR4E standard sample into a regression curve, the values of δ^2^H and δ^18^O were close to their standard values (δ^2^H = −49.2 ± 0.5‰, δ^18^O = −7.81 ± 0.15‰). It showed that the method can meet the requirement of accurate determination of a liquid water sample. The δ^18^O values of plant samples were corrected according to the method of Schultz et al. (2011). The leaf δ^13^C determination was carried out by Finnigan DELTAPlus XP stable isotope mass spectrometer (Thermo Electron Corp., Waltham, MA, United States). The value of plant leaf δ^13^C was calculated by the following formula: δ^13^C(‰) = (^13^C/ ^12^C_sample_-^13^C/ ^12^C_standard_)/(^13^C/ ^12^C_standard_) × 1000, in which ^13^C/ ^12^C_sample_ is the ^13^C/^12^C ratio of plant leaf samples, and ^13^C/ ^12^C_standard_ is the ^13^C/^12^C ratio of glycine in the determination process (Medina and Francisco, 1997).

Soil water content was determined by oven drying and weighing method (Wang and Chen, 2010). Soluble salt was analyzed by the gravimetric method (water/soil, 5:1) (Lu, 1999). Plant sample was digested by HNO_3_:HCl (3:1) and Na^+^ content in plant was determined by inductively coupled plasma atomic emission spectroscopy (ICP-AES, Thermo IRIS Intrepid II XDL, United States) according to the procedures of Emanuel et al. (2014). The total carbon (TC) and total nitrogen (TN) of soil and plant were determined by element analyzer (Vario EL III, Elementar, Germany) and total phosphorus (TP) was determined by molybdenum-antimony colorimetric method (Lu, 1999).

### Data Analysis

Excel 2010 (Microsoft Corp., Redmond, WA, United States) and SPSS19.0 (SPSS Inc., Chicago, IL, United States) were used to analyze the variance of the data. The contribution of potential water sources to xylem moisture of halophytes (*S. salsa*, *P. Communis*, and *L. bicolor*) was calculated by using IsoSource mixed model (Phillips and Gregg, 2003). The increment of model parameters was 1%, and the tolerance of mass balance was 0.05. The figures were plotted by Origin Pro 9.0 software.

## Results

### Water Content of Different Soil Layers

**FIGURE 1:**
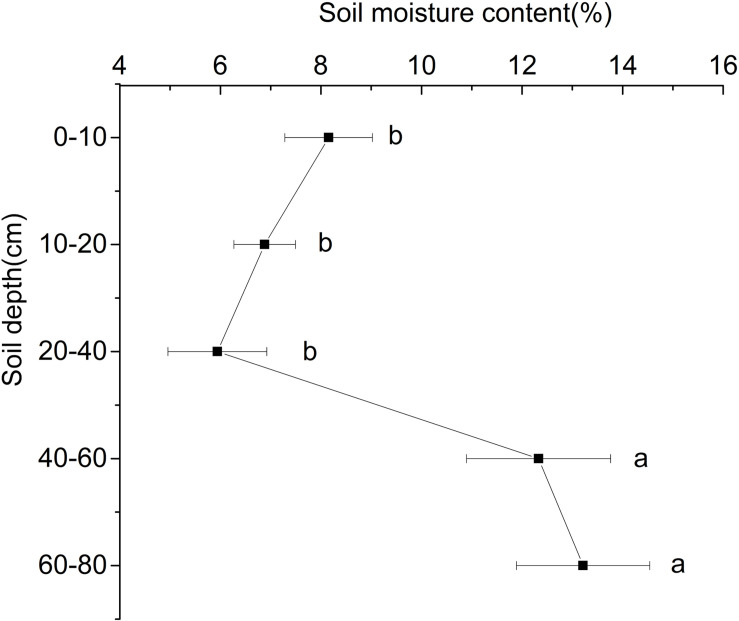
Water content of different soil layers of Shell Dike Island in the Yellow River Delta. Different letters mean a significant difference of 0.05.

With the increase of depth, the soil water content at the ridge of Shell Dike Island firstly decreased and then increased (Figure 1). Due to the influence of rainfall in July, the water content of the 0–10 cm soil layer was higher than that of the 10–40 cm soil layer, of which the 20–40 cm soil layer had the lowest water content, which was 5.94%. The water content of the 40–80 cm soil layer was significantly higher than that of the 0–40 cm soil layer, and the highest water content of the 60–80 cm soil layer was 13.21% (*P* < 0.05). Although the shallow soil water content was greatly affected by surface evaporation, the soil moisture content of the 0–10 cm soil layer was still higher than that of the 20–40 cm soil layer.

### Analysis of Soil Water Stable Isotope

**FIGURE 2:**
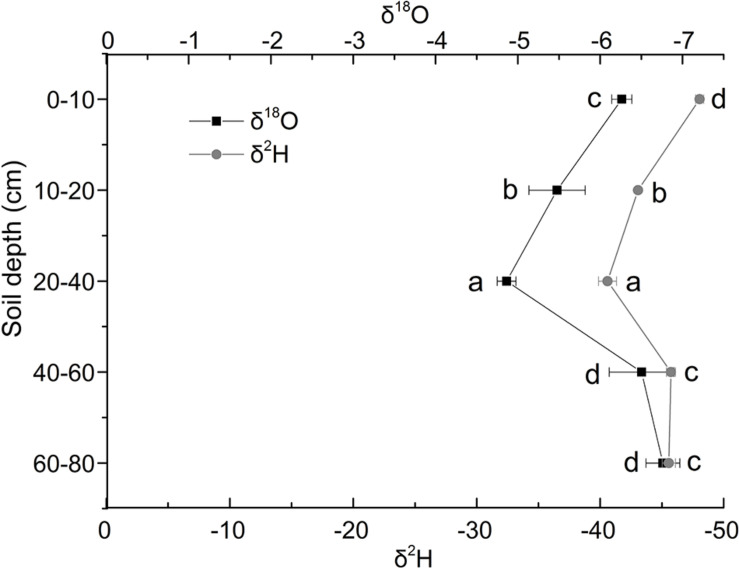
Characteristics of δ^18^O and δ^2^ H stable isotope of soil water in Shell Dike Island of the Yellow River Delta. Different letters mean a significant difference of 0.05.

The δ^18^O isotopic characteristics of soil water in Shell Dike Island of the Yellow River Delta were shown in Figure 2. In this study, the δ^18^O of soil water increased first and then decreased. It was the lowest in the 60–80 cm soil layer, and the highest in the 20–40 cm soil layer. The δ^18^O of soil water increased in the 0–40 cm soil layer, and decreased in the 40–80 cm soil layer. Besides, the δ^2^H (D) isotopic characteristics of soil water were shown in Figure 2. The δ^2^H value of soil water had the similar pattern with δ^18^O. It was the lowest in the 0–10 cm soil layer, and the highest in the 20–40 cm soil layer., the δ^2^H value of soil water increased in the 0–40 cm soil layer, and the δ^2^H value of soil water reduced continuously in the 40–80 cm soil layer.

### δ^18^O and δ^13^C Values for Different Halophytes

**FIGURE 3:**
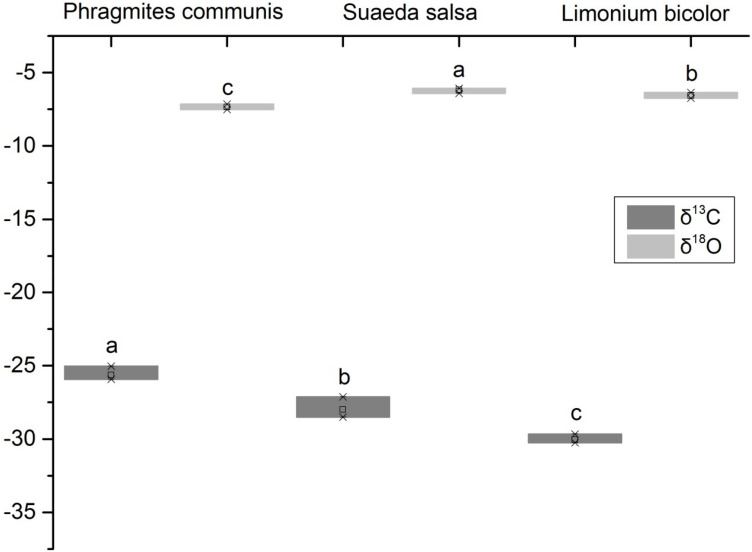
δ^18^O of xylem water and leaf δ^13^C values of different halophytes. Different letters mean a significant difference of 0.05.

It showed that the δ^18^O values of xylem water of three halophytes were significantly different (Figure 3). The δ^18^O value of *S. salsa* was the highest, followed by *L. bicolor*, and *P. communis* was the lowest. The leaf δ^13^C value of *P. communis* was relatively higher than that of *S. salsa* and *L. bicolor*. The δ^13^C values of the three halophytes ranged from −25.63 to −30.05‰. It showed that the water use efficiency of *P. communis* was the highest, followed by *S. salsa*, and *L. bicolor* was the lowest. The results showed that there were significant differences in water use efficiency among three halophytes. The δ^13^C value of *P. communis* (−25.63‰) was significantly high compared with the global survey (−28.74‰). *S. salsa* (−28.01‰) was close to the global value, and *L. bicolor* (−30.05‰) was significantly low compared with it. In general, the average carbon isotope value of three halophytes was −27.90‰, which was significantly high (*P* < 0.05).

### Water Use of Different Halophytes

**FIGURE 4:**
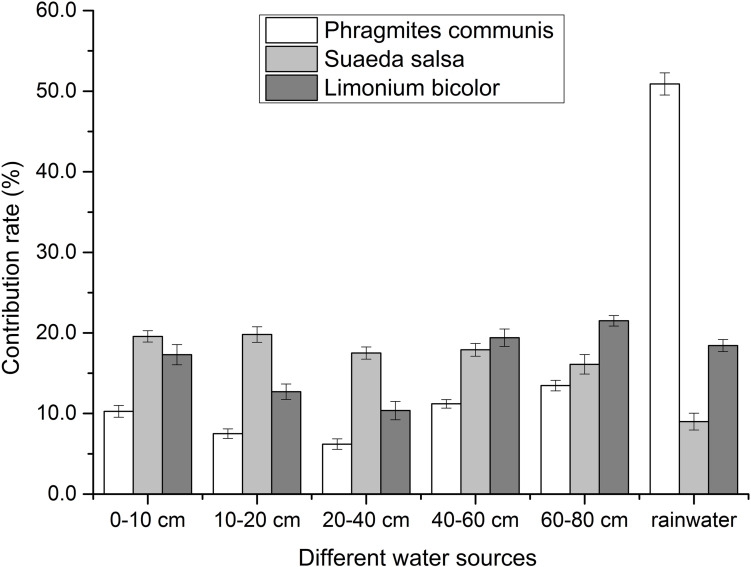
Contribution of different water sources to different halophytes.

The water use of different halophytes was shown in Figure 4. It showed that *P. communis* mostly absorbed and utilized the rainwater, the contribution of rainwater was 50.9%. The total contribution of soil water was 49%. With the depth of soil water, the contribution of soil water to *P. communis* decreased first and then increased. The lowest contribution of soil water to *P. communis* was 6.2% in the depth of 20–40 cm, and the highest was 13.4% in the depth of 60–80 cm.

In each layer, *S. salsa* mainly absorbed the water from soil water. The total contribution of soil water to *S. salsa* was 90.9%. With the increase of soil water depth, the contribution of soil water to *S. salsa* decreased. The lowest contribution of soil water to *S. salsa* was 16.1% in the depth of 60–80 cm. The highest contribution of soil water to *S. salsa* was 19.8% in the depth of 10–20 cm, and the contribution of rainwater was 9%.

The absorption and utilization of rainwater by *L. bicolor* was 18.5%, while the contribution of soil water to *L. bicolor* was 81.4% in all layers. So the water absorbed and used by *L. bicolor* mainly came from soil water. With the increase of soil water depth, the contribution of soil water in different depths decreased first and then increased. In different depths of soil water, the highest contribution was 21.5% at in the depth of 60–80 cm, and the lowest contribution was 10.5% in the depth of 20–40 cm.

### Salt Content in Different Soil Layers

**FIGURE 5:**
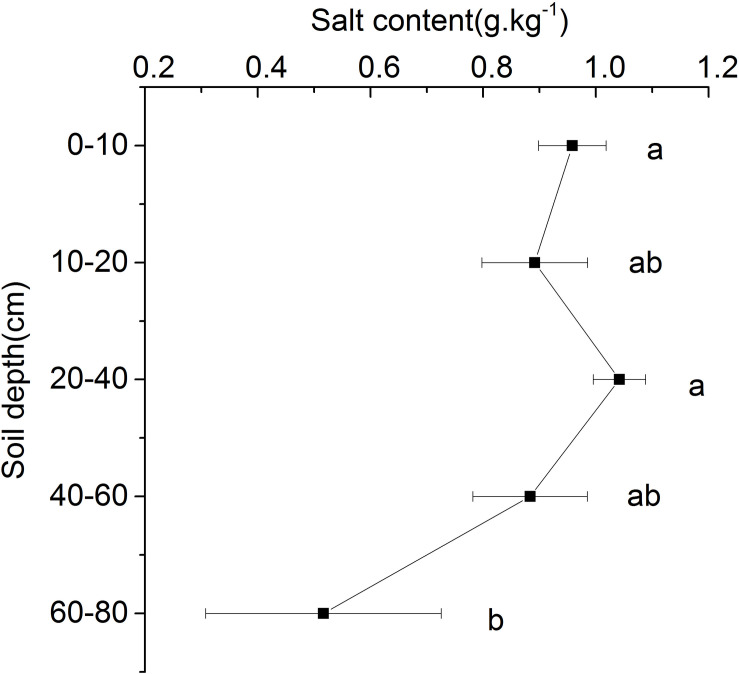
Salt content in different soil layers. Different letters mean a significant difference of 0.05.

The results showed that the salt content of each soil layer in the beach ridge of Shell Island ranged from 0.52 to 1.04 g.kg^–1^. With the increase of soil depth, there was no significant difference in salt content between the 0 and 60 cm soil layers, while the salt content of the 60–80 cm soil layer was significantly lower than that of the 0–60 cm soil layer (Figure 5).

### Na^+^ Content in Plant Tissues

**FIGURE 6:**
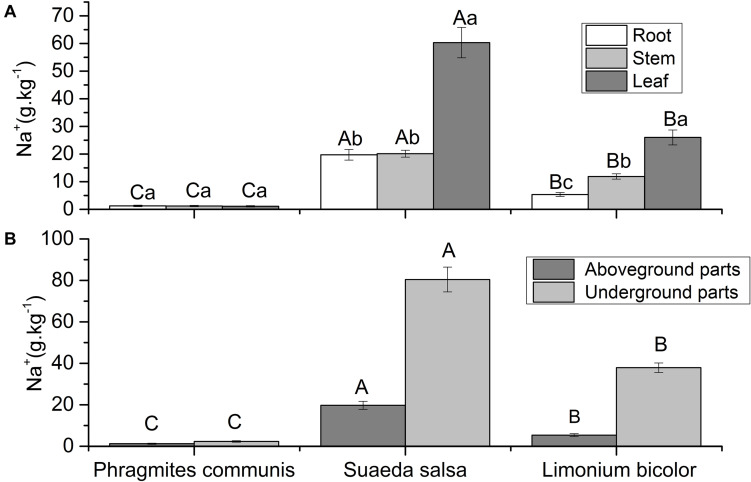
Comparison of Na^+^ contents in roots, stems, leaves **(A)** and aboveground, underground parts **(B)** of three different halophytes. Significant differences between the same plant are marked with lowercase letters, and significant differences among different plant species are marked with capital letters.

The Na^+^ content in the same organs of different plants and different organs of the same plant were different (Figure 6). The Na^+^ content in different tissue parts of *P. communis* showed no significant difference, and was significantly lower than *S. salsa* and *L. Bicolor*. The Na^+^content in leaves of *S. salsa* was significantly higher than that in roots and stems. The Na^+^contents in roots, stems, and leaves of *S. salsa* were significantly higher than those of the other two halophytes. The Na^+^ content in different tissues of *L. bicolor* was significantly different, with the order of leaf > stem > root. The Na^+^ content in different halophytes was significantly different, which was *S. salsa* > *L. bicolor* > *P. communis*. The results showed that the Na^+^ content in the aboveground and underground parts of different halophytes was significantly different, with an order of *S. salsa* > *L. bicolor* > *P. communis*.

### Stoichiometry Characteristics in Different Soil Layers

**FIGURE 7:**
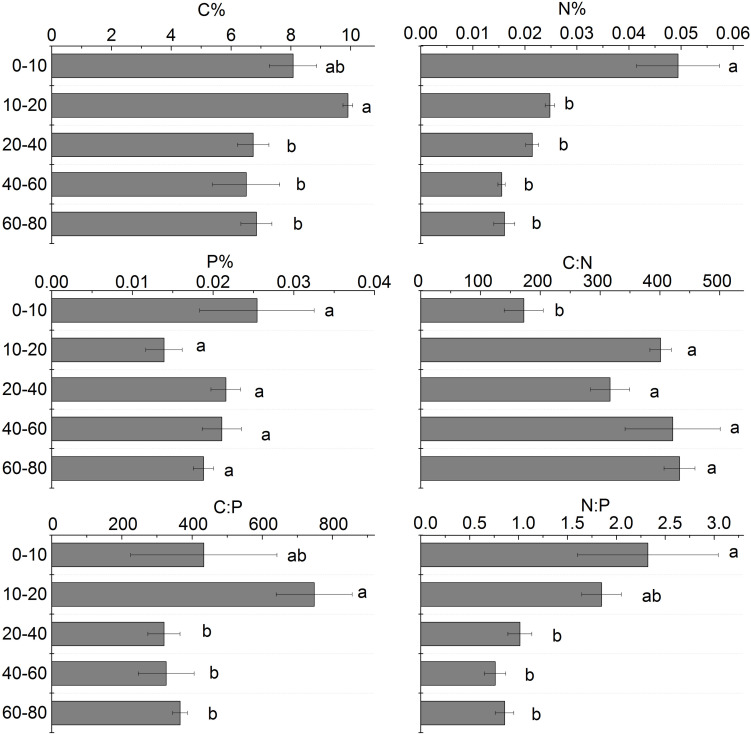
Stoichiometric analysis of different soil layers. Different letters mean a significant difference of 0.05.

The soil C content in different soil layers ranged from 6.50 to 9.91%. The soil C content generally decreased with depth and the greatest content existed in the 10–20 cm soil layer. The soil N content in different soil layers ranged from 0.016 to 0.049%. From 0 to 80 cm soil layer, the soil N content had a similar pattern with C content. The soil N content of the 0–10 cm soil layer was significantly higher than that of the 20–80 cm soil layers. The soil P content in different soil layers ranged from 0.16 to 0.49 g.kg^–1^. The soil P content was no significantly different in the 0 to 80 cm soil layers (Figure 7).

C:N in different soil layers increased with the increase of soil depth, and the difference between 10 and 80 cm soil layer was not significant, but it was significantly higher than that of the 0–10 cm soil layer; C:P increased first and then decreased with the increase of soil depth, and C:P in the 20–80 cm soil layer was significantly lower than that of 0–20 cm soil layer (Figure 7).

### Stoichiometry Characteristics in Different Halophytes

**FIGURE 8:**
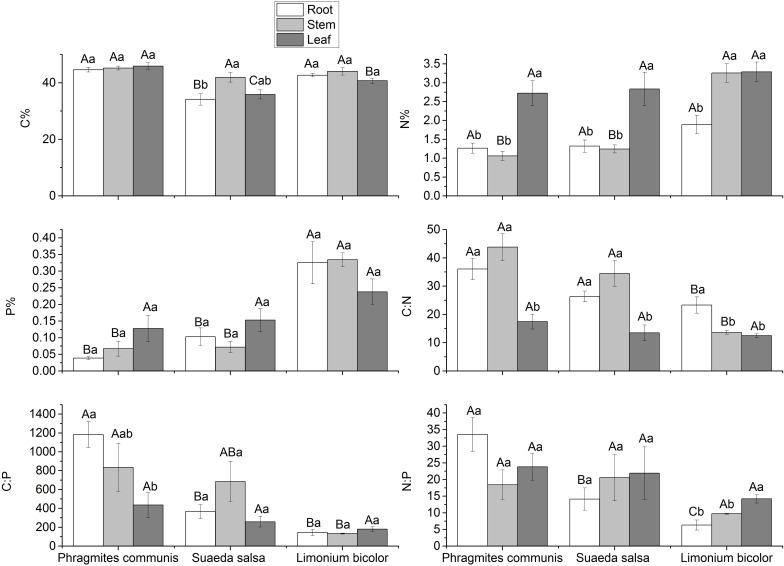
Stoichiometry of different tissues of different halophytes. Significant differences between the same plant are marked with lowercase letters, and significant differences among different plant species are marked with capital letters.

The C:N:P stoichiometric characteristics in three halophytes were significantly different (Figure 8). Compared with the same tissue of different halophytes, the C content in roots was *P. communis* > *L. bicolor* > *S. salsa*, but there was no significant difference in stems, while in leaves was *P. communis* > *L. bicolor* > *S. salsa*. The N content showed no significant difference in the roots and leaves of the three halophytes. The difference of N content existed in the stem, which was *L. bicolor* > *S. salsa* = *P. communis*. The results showed that P content in the roots was as follows: *L. bicolor* > *S. salsa* = *P. communis*. The P content in stems was similar to the roots in three halophytes, but there was no significant difference in leaves. In general, P content in tissues of *L. bicolor*, especially in roots and stems, was significantly higher than that in *S. salsa* and *P. communis*. C:N in roots and stems was *P. communis* > *S. salsa* > *L. bicolor*. The C:P in roots was *P. communis* > *S. salsa* = *L. bicolor*, *P. communis* > *S. salsa* > *L. bicolor* in stems, but there was no significant difference among the three in leaves. N:P in roots was *P. communis* > *L. bicolor* > *S. salsa*, but there was no significant difference in stems and leaves.

### Correlations Among the Indexes of Different Halophytes

The correlations between leaf stoichiometry and Na^+^ content in leaves of three halophytes were significantly different (Table 1). Specifically, N content and P content in *P. communis* leaves were significantly positively correlated, while C content and N:P in leaves were significantly positively correlated with Na^+^ content (correlation coefficient was 0.685, 0.698, respectively), The correlation index of *S. salsa* was the least among the three halophytes, and its stoichiometry characteristics had no significant correlation with Na^+^ content. N content and C:N in *L. bicolor* were significantly positively correlated (0.789) and negatively correlated with Na^+^ content (−0.841), in addition, the C content was significantly positively correlated with N content and P content.

**TABLE 1:** Correlations between the leaf stoichiometry characteristics, Na^+^ content, and δ^13^C indexes of three different species of halophytes.

**Index**	***Phragmites communis***						***Suaeda salsa***					***Limonium bicolor***						
	**P**	**C:N**	**C:P**	**N:P**	**Na^+^**	**δ ^13^C**	**C:N**	**C:P**	**N:P**	**Na^+^**	**δ ^13^C**	**N**	**P**	**C:N**	**C:P**	**N:P**	**Na^+^**	**δ ^13^C**
C	–0.466	0.731*	0.682*	0.480	0.685*	–0.055	0.662	0.314	0.091	–0.039	–0.364	0.695*	0.742*	–0.508	–0.650	–0.027	0.226	–0.212
N	0.674*	−0.967**	−0.786*	–0.490	–0.135	0.509	−0.826**	0.220	0.535	0.645	0.477	1	0.338	−0.945**	–0.343	0.596	0.789*	–0.108
P	1	–0.666	−0.918**	−0.939**	–0.598	0.084	0.198	−0.836**	−0.845**	–0.492	–0.340		1	–0.187	−0.971**	–0.553	0.084	–0.322
C:N		1	0.830**	0.517	0.271	–0.360	1	–0.057	–0.401	–0.540	–0.545			1	0.208	−0.683*	−0.841**	–0.083
C:P			1	0.900**	0.576	–0.131		1	0.925**	0.472	0.181				1	0.528	–0.170	0.321
N:P				1	0.698*	0.055			1	0.619	0.423					1	0.642	0.188
Na					1	0.294				1	0.084						1	0.046
δ^13^C						1					1							1

N = 3. *P < 0.05. **P < 0.01.

The C content and C:N in leaves of three halophytes were consistent. Although the above correlations did not reach the significant difference level, they were all negatively correlated with δ^13^C index. The correlations between N:P and Na^+^ content and δ^13^C were all positively correlated, but the correlations were not significant (Table 1).

### Correlations Between Different Halophytes and Different Soil Layers

**FIGURE 9:**
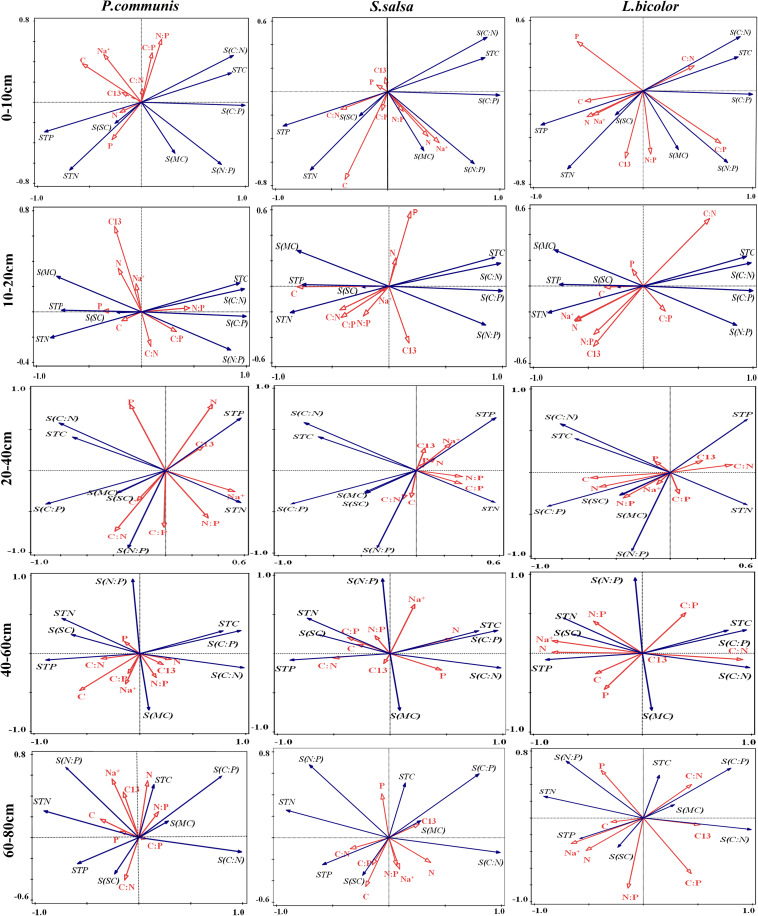
RDA analysis of the relationships between three halophytes and environmental factors in different soil layers. TC, Plant total carbon; TN, Plant total nitrogen; TP, Plant total phosphorus; C:N, Plant C/N ratio; C:P, Plant C/P ratio; N:P, Plant N/P ratio; Na^+^, Sodium ion of plant; C^13^, Carbon isotope of plant; STC, Soil total carbon; STN, Soil total nitrogen; STP, Soil total phosphorus; S(C:N), Soil C/N ratio; S(C:P), Soil C/P ratio; S(N:P), Soil N/P ratio; S(SC), Salt content of soil; S(MC), Moisture content of soil.

The relationships between environmental factors and plant factors in different soil layers were analyzed by Redundancy analysis (RDA). In *P. communis*, the correlation between plants and soil layers was as follows: In the 10–20 cm soil layer, leaf P content and N:P were positively correlated and negatively correlated with soil salt content, respectively. In the 20–40 cm soil layer, the significant correlation indexes between plant and soil were significantly increased, leaf C content was significantly positively correlated with soil water content, leaf N content was positively and negatively correlated with soil P content and N:P, respectively; leaf P content was positively and negatively correlated with soil C:N and N:P; leaf C:N was positively correlated with soil N:P. In the 40–60 cm soil layer, there was a significant negative correlation between leaf C content and soil C:P, and leaf C:P, N:P, leaf Na^+^ content were significantly positively correlated with soil water content. In the 60–80 cm soil layer, leaf C:N was positively correlated with soil salt content (Figure 9).

In *S. salsa*, with the increase of soil depth, the correlation between the indexes of *S. salsa* leaves and soil decreased (Figure 9). In the 0–10 cm soil layer, leaf C content was positively correlated with soil N content, and leaf C content was negatively correlated with soil C:N. In the 10–20 cm soil layer, the leaf C content was significantly negatively correlated with the soil C content, C:N, and C:P. Leaf C:P and soil N content was significantly positively correlated, leaf Na^+^ content was significantly negatively correlated with soil water content, and leaf C:N was significantly positively correlated with soil salt content in the 20–80 cm soil layer.

In *L. bicolor*, leaf C:P was positively correlated with soil C:P and N:P in the 0–10 cm soil layer. In the 10–20 cm, leaf N content was positively correlated with soil water content, leaf C:N was positively correlated with soil C:N. In the 20–40 cm, leaf N content was positively correlated with soil C content. In the 40–60 cm, leaf N content was positively correlated with soil N content, P content and soil salt content, and leaf Na^+^ content was significantly positively correlated with soil N content, P content and salt content (Figure 9).

## Discussion

### Water Use Patterns and Water Use Efficiencies of Different Halophytes

Plant water sources will be adjusted according to water supply (Duan et al., 2008). There were apparent variations in the xylem water δ^18^O values of different plants (Cui et al., 2021). In this study, the xylem water δ^18^O of three halophytes species were significantly different, which indicated that there were interspecific differences in the water absorption. The water source of the plants in the same habitat may be diverse, and this diversity is usually of positive significance for the coexistence of plants (Min et al., 2019). There are obvious differences in rainwater utilization rate among the three halophytes. Compared with the other two types of halophytes, *P. communis* has the highest rainwater utilization rate, which reflects its advantage in freshwater utilization. Soil water is the source of water that plants can use directly. The contribution of soil water to *S. salsa* was the highest when the soil depth was 10–20 cm. For *P. communis* and *L. bicolor*, the highest contributions of soil water were both in the depth of 60–80 cm, which indicated that the latter two mainly used the deep soil water. The above results may be attributed to the root distribution depth and spatial distribution structure of three halophytes (Nie et al., 2011; Ren et al., 2017). There is a characteristic of water uptake by plant roots, that is, the dependence of plants on different water content changes with the availability of this water source (Dawson and Pate, 1996; Mccole and Stern, 2007; Guo et al., 2018).

δ^13^C can comprehensively reflect the physiological and external environmental characteristics of various factors affecting the relationship between carbon and water during vegetation growth. Many studies have shown that δ^13^C is highly correlated with plant WUE. Therefore, leaf δ^13^C can be used to reflect the WUE characteristics of different plant species (Farquhar et al., 1982; Stokes et al., 2010; Baruch, 2011). In this study, the value of δ^13^C in leaves of *P. communis* is the highest, which indicates that *P. communis* has the highest utilization efficiency of water resources form habitat. The water use efficiency of *L. bicolor* is the lowest, which indicates that it belongs to the profligate consumption of soil water (Quiroga et al., 2013; Ding et al., 2016). The reason may be related to the different sources of water use of the three halophytes.

### Correlations of Water, Salt, and Stoichiometry in Different Halophytes

C, N, and P are the essential elements of organisms, and have strong interactions in biological function (Sterner and Elser, 2002). Plant stoichiometry varies mainly with environment and taxonomic affiliation (Zhang et al., 2016). In this study, C content in roots and leaves of *P. communis* were highest among the three halophytes, indicating that *P. communis* has a stronger ability of carbon fixation by photosynthesis. N content in stems, and P content in roots and stems of *L. bicolor* were highest among the three halophytes, which showed that *L. bicolor* has a higher nitrogen and phosphorus absorption efficiency. C:N and C:P, being important physiological indices, can also reflect the growth rate of plants (Frost et al., 2005; Wang N. et al., 2018). C:N and C:P in roots and stems of *P. communis* were highest among the three halophytes, indicating that construction efficiency of roots and stems in *P. communis* is higher than *S. salsa* and *L. bicolor*.

The changes of contents of C, N, and P must be accompanied with the transportation of salt ions in halophytes. It is reported that salt ions and N can promote each other. It is speculated that N can effectively promote salt ion absorption in halophytes and contribute to the improvement of halophytes in a saline-alkali habitat (Shao et al., 2005; Yong et al., 2016). In the study, C content and N:P in leaves of *P. communis* were significantly positively correlated with Na^+^, and the N content of leaves in *L. bicolor* was positively correlated with Na^+^. The findings are consistent with previous research. While N content of leaves in *S. salsa* had no significant correlation with Na^+^, which needs to be further studied. The correlation between the stoichiometry characteristics and Na^+^ in the leaves of the three halophytes is consistent with the salt tolerance mechanism of the three halophytes to a great extent.

Although there was no significant correlation between the δ^13^C values of the three halophytes and their leaf C content and C:N, they all showed the same trend. This indicates that water use efficiency of leaves has an influence on the stoichiometric characteristics of C:N:P in leaves. As pointed out by Flexas et al. (2016), the increase of WUE at the leaf level does not necessarily promote the increase of WUE at the whole plant level. This is because the favorable factors of improving WUE at the leaf level may be offset by other factors, which are not completely independent of WUE (Flexas et al., 2016). For instance, the growth of plant leaves causes a large shading area, and the increase of plant respiration may lead to the increase of carbon catalysis, etc. (Thomas et al., 2013), which is consistent with our result that the δ^13^C value and C content of three halophytes are negatively correlated.

The long-term nitrogen use efficiency (NUE) of plant leaves is expressed by leaf C:N (Livingston et al., 1999), which provides a broad view of organic matter source. This parameter characterizes the efficiency of nitrogen distribution and utilization for carbon acquisition and assimilation (Hedges et al., 1986). Studies have shown that the WUE and NUE of different dominant plants in the main forest ecosystems in the north-south transect of eastern China are significantly negatively correlated, indicating that plants with higher water use efficiency tend to have lower nitrogen use efficiency in natural ecosystems (Field et al., 1983; Patterson et al., 1997; Sheng et al., 2011). In this study, the characteristic parameters δ^13^C of WUE were all negatively correlated with C:N in three halophytes. This result is consistent with the above research and it is in line with the relevant conclusion that plants can not optimize the use of water and nitrogen at the same time in natural ecosystems, and its utilization strategy is to make efficient use of one resource at the expense of another (Salazar-Tortosa et al., 2018).

### Correlations of Water, Salt, and Stoichiometry in Plant and Soil, Respectively

Water, nutrients, and salt are the important resources and conditions that affect the growth and development of plants. How to optimally utilize and integrate water, salt, and nutrients is related to the varying behaviors in different plants. Soil salinity is one of the main factors which contributes to plant δ^13^C. The analysis of the correlation between plant δ^13^C and soil salinity can provide an important reference for judging the physiological and ecological adaptability of plants to salt (Costantini et al., 2010). In this study, the correlation between Na^+^ content and δ^13^C in leaves of three halophytes did not reach significant level, but all showed positive correlation trend. Farquhar et al. (1989) have already suggested that the δ^13^C value of plants increased with the increase of salinity. Many studies have also shown that there is a positive correlation between the δ^13^C value of plant leaves and soil salinity, whether they are halophytes (such as *Mesembryanthemum crystallinum*, *Puccinellia nuttalliana*, *Avicennia marina*, etc.) (Guy and Reid, 1986; Winter and Holtum, 2005; Ladd and Sachs, 2013) or non-halophytes (wheat, safflower, tomato, etc.) (Yousfi et al., 2012; Amor and Francisco, 2013; Hussain and Al-Dakheel, 2018). The reason of soil salinity affecting plant δ^13^C is that salt environments cause some changes in plant physiological activities (such as CO_2_ diffusion, transfer, or photosynthetic rate) (Farquhar et al., 1982; Flanagan and Jefferies, 1989).

The C:N:P ratio in soil directly reflects soil fertility and plant nutritional status (Elser et al., 2010). A fundamental study found positive correlations between plant leaf and soil nutrients in 1900 plant species across China (Han et al., 2011). Regression analyses showed that soil N:P ratios were significantly correlated with leaf N:P ratios in subtropical Eucalyptus plantations (Fan et al., 2015). Positive relationships between soil C:N:P and leaf nutrient ratios in Australia’s major native vegetation ecosystems have also been observed (Bui and Henderson, 2013). The C:P and N:P of soil were positively correlated with plant tissues of black locust plants on the Loess Plateau (Cao and Chen, 2017). In this study, leaf C:N of *P. communis* was positively correlated with soil N:P. Leaf C:P of *L. bicolor* was positively correlated with soil C:P and N:P, leaf C:N was positively correlated with soil C:N. leaf C:P of *S. salsa* was positively correlated with soil N. The results show that the stoichiometry between soil and plant were influenced not only by soil factors, but also by plant physiological metabolism.

Considering the fact that these soil-sourced factors changed variously, their effect on leaf stoichiometry is complex (Wang et al., 2015). In this study, leaf C:N of *P. communis* was positively correlated with soil salt content, and leaf C:P and N:P were significantly positively correlated with soil water content. Leaf C:N ratio of *S. salsa* was significantly positively correlated with soil salt content, and leaf N of *L. bicolor* was positively correlated with soil water content. The results show that soil salinity and soil water are also important factors for plant stoichiometry in coastal ecosystems.

In the same habitat, there is competition among different plants in resources, how to reasonably obtain the related resources and minimize the restriction of a certain environmental factor on its growth is an important survival strategy for plants (Barot et al., 2014; Sebastien et al., 2016; Falster et al., 2017). Based on the correlation analysis of water sources, stoichiometry, and the salt content between soil and plant, the survival strategies of three types of halophytes were found. The soil water contribution of the 60–80 cm soil layer to *P. communis* is the largest and the soil layer salt content is the lowest, which indicates that *P. communis* prefer to use the soil water with low salt concentration. However, the closest correlation of C:N:P stoichiometry between plant leaves and soil is in the 20–40 cm soil layer. *S. salsa* and *L. bicolor* not only use the soil water of the soil layer with a low salt content, but also have high utilization of the soil water of the soil layer with a relatively high salt concentration. The soil water contribution of the 10–20 cm soil layer to *S. salsa* is the highest, and the closest correlation of C:N:P stoichiometry between plant leaves and soil is also in the 10–20 cm soil layer. The soil water contribution of the 40–60 cm soil layer to *L. bicolor* is the highest, and the closest correlation of C:N:P stoichiometry between plant leaves and soil is also in the 40–60 cm soil layer. This indicates that there is spatial consistency of soil in water and nutrient utilization of *S. salsa* and *L. bicolor*. The differences of correlation patterns between the three halophytes and each soil layer are maybe caused by ecophysiological strategies of different halophytes, which can make the most positive and effective response to the heterogeneity of the surrounding soil environment, so as to achieve the best resource utilization efficiency of water, salt, and nutrients.

## Conclusion

Our research indicated that there were significant differences in water sources and water contribution from different soil depth among the three halophytes. *P. communis* showed certain advantages in the above water adaptability, while *L. bicolor* was a relatively water consuming plant. The differences of Na^+^ content in different tissues of three halophytes showed that *S. salsa* had a stronger salt tolerance than *L. bicolor* and *P. communis*. In terms of nutrient adaptability, *L. bicolor* showed higher N and P uptake, while *P. communis* showed a higher construction efficiency of roots and stems. Moreover, the effect of Na^+^ on the stoichiometry of *L. bicolor* and *P. communis* was greater than that of *S. salsa*. The correlations between soil depth and different halophytes indicated that the adaptation strategies are different. The soil layers which were closely related to the water and nutrient use of *P. communis* were not coincidental, while the water and nutrient sources of the *S. salsa* and *L. bicolor* had spatial consistency. Therefore, in the process of vegetation restoration in the Yellow River Delta, it is possible to achieve better results by allocating different halophytes according to their adaptability and the heterogeneity of soil vertical structure.

## Data Availability Statement

The original contributions presented in the study are included in the article/supplementary material, further inquiries can be directed to the corresponding author.

## Author Contributions

JS designed, managed, and supervised the whole project. TL analyzed the experimental data and wrote the manuscript. ZF carried out field sample collection and experimental operation. All authors read and approved the manuscript.

## Conflict of Interest

The authors declare that the research was conducted in the absence of any commercial or financial relationships that could be construed as a potential conflict of interest.
